# Supplementary material for: Emergency FMD Serotype O Vaccines Protect Cattle against Heterologous Challenge with a Variant Foot-and-Mouth Disease Virus from the O/ME-SA/Ind2001 Lineage
Source: Vaccines (Basel). 2021 Sep 29;9(10):1110. doi: 10.3390/vaccines9101110 (PMC8537456; doi:10.3390/vaccines9101110)
Supplement: Supplementary file 1 [file vaccines-09-01110-s001.zip › vaccines-1369748-supplementary.pdf]

Supplementary Table S1. Homologous and heterologous post vaccination neutralising antibody titres (21 or 7 days post vaccination) and clinical observations at 8 days post challenge in cattle vaccinated with a O-3039 + O1 Manisa combination vaccine (Combo) or O-3039 monovalent vaccine and an unvaccinated control group (UVC). Titres are expressed as log_{10} values at the time of challenge. Combo-21 and O-3039-21 groups were challenged on 21 dpv while Combo-7 and O-3039-7 groups were challenged on 7 dpv. All sera were tested against O1 Manisa, O-3039 and O-ALG virus. Titres ≥1.20 log_{10} are considered positive and are highlighted in bold. FMD Lesions: + - protected; LF – Left forelimb; BF – Both forelimbs; BH – Both hindlimbs

Groups	Animal ID	O-3039	O1 Manisa	O-ALG	Clinical Status
Combo-21	9658	**2.40**	**2.10**	**2.40**	+
	9659	**2.40**	**2.10**	**2.40**	+
	9660	**2.40**	**1.95**	**2.40**	+
	9661	**2.25**	**2.10**	**2.40**	+
	9662	**3.00**	**2.40**	**2.10**	+
O3039-21	9663	**3.00**	**1.95**	**2.85**	+
	9664	**3.00**	**2.10**	**2.10**	+
	9665	**2.70**	**2.25**	**2.40**	+
	9666	**2.40**	**1.95**	**2.40**	+
	9667	**2.70**	**1.80**	**2.10**	+
Combo-7	9668	**2.25**	0.90	**1.35**	+
	9669	1.05	0.75	0.60	+
	9670	**2.40**	**1.50**	**2.10**	+
	9671	**1.95**	**1.50**	**1.35**	+
	9672	**2.10**	**1.80**	**1.65**	Not protected; BF
O3039-7	9673	**2.10**	**1.35**	**2.10**	+
	9674	**2.10**	**1.80**	**1.35**	Not protected; BH
	9675	**1.95**	0.75	**1.80**	+
	9676	**2.10**	1.05	**2.40**	+
	9677	**1.80**	**1.50**	1.05	Not protected; BF, RH
UVC	9678	0.30	0.30	0.30	Generalised; LF, BH
	9679	0.30	0.30	0.45	Generalised; BF, BH
	9680	0.30	0.30	0.30	Generalised; BF, BH

Supplementary Text ST-1

###### vaccine groups against challenge #########

> protection.vac<-matrix(c(5,0,5,0,4,1,3,2), byrow=T, ncol=2, dimnames=list(c("Combo-21","O3039-21","Combo-7","O3039-7"), c("Protected","Not protected")))

> protection.vac

	Protected	Not protected
Combo-21	5	0
O3039-21	5	0
Combo-7	4	1
O3039-7	3	2

> fisher.test(protection.vac)

Fisher's Exact Test for Count Data

data: protection.vac

p-value = 0.5614

alternative hypothesis: two.sided

Supplementary Text ST-2

###### vaccinated vs control against challenge #########

> pr<-matrix(c(3,17,3,0),byrow=T,ncol=2,dimnames=list(c("vaccinated","control"),c("non-protected","Protected")))

> pr

	non-protected	Protected
vaccinated	3	17
control	3	0

> fisher.test(pr)

Fisher's Exact Test for Count Data

data: pr

p-value = 0.01129

alternative hypothesis: true odds ratio is not equal to 1

95 percent confidence interval: 0.0000000 0.6866581

sample estimates: odds ratio = 0

###### day post vaccination against challenge #########

> table(prot1,day)

	day	
prot1	21	7
0	0	3
1	10	7

> fisher.test(table(prot1,day))

Fisher's Exact Test for Count Data

data: table(prot1, day)

p-value = 0.2105

alternative hypothesis: true odds ratio is not equal to 1

95 percent confidence interval: 0.000000 2.255229

sample estimates: odds ratio = 0

Supplementary Text ST-3

####### body temperature #######

```
> body<-read.csv("bodytemp.csv",sep=';')
> attach(body)
> summary(body)
> f0<-lmer(temp~1+(1|animal))
> f1<-lmer(temp~group+(1|animal))
> f2<-lmer(temp~factor(dpi)+(1|animal))
> f3<-lmer(temp~prot+(1|animal))
> AIC(f0,f1,f2,f3)
```

	df	AIC
f0	3	947.4081
f1	7	966.3614
f2	36	476.3922
f3	4	948.8909

```
> f4<-lmer(temp~factor(dpi)+group+(1|animal))
> f5<-lmer(temp~factor(dpi)+prot+(1|animal))
> AIC(f2,f4,f5)
```

	df	AIC
f2	36	476.3922
f4	40	495.3455
f5	37	477.8750

```
> summary(f2)
```

Linear mixed model fit by REML ['lmerMod']
Formula: temp ~ factor(dpi) + (1 | animal)
REML criterion at convergence: 404.4

Min	1Q	Median	3Q	Max
-5.079	-0.3955	0.0407	0.415	5.7126

Random effects:
Number of obs: 782, groups: animal, 23

Groups	Name	Variance	Std.Dev.
animal	(Intercept)	0.009398	0.09694
Residual		0.083230	0.28850

Fixed effects:

	Estimate	Std. Error	t value
(Intercept)	3.89E+01	6.35E-02	612.904
factor(dpi)1	1.17E+00	8.51E-02	13.748
factor(dpi)2	1.27E+00	8.51E-02	14.974
factor(dpi)3	6.83E-01	8.51E-02	8.024
factor(dpi)4	3.70E-01	8.51E-02	4.344
factor(dpi)5	-7.39E-02	8.51E-02	-0.869
factor(dpi)6	-1.26E-01	8.51E-02	-1.482
factor(dpi)7	-1.00E-01	8.51E-02	-1.175
factor(dpi)8	8.70E-03	8.51E-02	0.102
factor(dpi)9	-5.22E-02	8.51E-02	-0.613
factor(dpi)10	-2.61E-02	8.51E-02	-0.307
factor(dpi)11	-9.57E-02	8.51E-02	-1.124
factor(dpi)12	-7.83E-02	8.51E-02	-0.920
factor(dpi)13	8.44E-13	8.51E-02	0.000
factor(dpi)14	-8.70E-03	8.51E-02	-0.102
factor(dpi)15	8.59E-13	8.51E-02	0.000
factor(dpi)16	7.83E-02	8.51E-02	0.920
factor(dpi)17	-4.78E-02	8.51E-02	-0.562
factor(dpi)18	-6.96E-02	8.51E-02	-0.818
factor(dpi)19	-1.26E-01	8.51E-02	-1.482
factor(dpi)20	-9.13E-02	8.51E-02	-1.073
factor(dpi)21	-3.91E-02	8.51E-02	-0.460
factor(dpi)22	-3.04E-02	8.51E-02	-0.358

factor(dpi)23	-2.17E-02	8.51E-02	-0.256
factor(dpi)24	-1.74E-02	8.51E-02	-0.204
factor(dpi)25	-4.78E-02	8.51E-02	-0.562
factor(dpi)26	-8.70E-02	8.51E-02	-1.022
factor(dpi)27	-1.44E-01	8.51E-02	-1.687
factor(dpi)28	2.17E-02	8.51E-02	0.256
factor(dpi)29	-8.70E-02	8.51E-02	-1.022
factor(dpi)30	-7.39E-02	8.51E-02	-0.869
factor(dpi)31	5.65E-02	8.51E-02	0.664
factor(dpi)32	-6.96E-02	8.51E-02	-0.818
factor(dpi)33	-1.30E-01	8.51E-02	-1.533

Correlation matrix not shown by default, as p = 34 > 12.
Use print(x, correlation=TRUE) or vcov(x) if you need it

```
> for (i in 2:15){
+ f0<-lmer(temp~1+(1|animal),body[dpi<i,])
+ f1<-lmer(temp~group+(1|animal),body[dpi<i,])
+ f2<-lmer(temp~factor(dpi)+(1|animal),body[dpi<i,])
+ f3<-lmer(temp~factor(dpi)+group+(1|animal),body[dpi<i,])
+ print(i)
+ print(AIC(f0,f1,f2,f3)) }
```

boundary (singular) fit: see? is Singular
boundary (singular) fit: see? is Singular
boundary (singular) fit: see? is Singular
[1] 2

	df	AIC
f0	3	120.07876
f1	7	126.15642
f2	4	91.16423
f3	8	96.65586

boundary (singular) fit: see? is Singular
boundary (singular) fit: see? is Singular
[1] 3

	df	AIC
f0	3	182.25030
f1	7	187.17900
f2	5	146.08060
f3	9	149.69970

boundary (singular) fit: see? is Singular
[1] 4

f0	3	230.00020
f1	7	232.15120
f2	6	188.15440
f3	10	190.22030

[1] 5

	df	AIC
f0	3	278.70060
f1	7	279.85200
f2	7	228.93650
f3	11	230.08790

boundary (singular) fit: see? is Singular
[1] 6

	df	AIC
f0	3	334.35490

f1	7	335.55210
f2	8	260.76850
f3	12	261.92160

boundary (singular) fit: see? is Singular
[1] 7

	df	AIC
f0	3	383.77290
f1	7	387.45840
f2	9	287.34250
f3	13	290.85250

boundary (singular) fit: see? is Singular
[1] 8

	df	AIC
f0	3	427.19550
f1	7	432.62610
f2	10	309.84600
f3	14	315.13510

boundary (singular) fit: see? is Singular
[1] 9

	df	AIC
f0	3	463.94030
f1	7	470.98280
f2	11	331.38000
f3	15	338.38030

boundary (singular) fit: see? is Singular
[1] 10

	df	AIC
f0	3	499.34070
f1	7	506.30530
f2	12	348.50320
f3	16	355.41730

boundary (singular) fit: see? is Singular
[1] 11

	df	AIC
f0	3	532.43040
f1	7	539.84640
f2	13	367.52820
f3	17	374.84470

boundary (singular) fit: see? is Singular
[1] 12

	df	AIC
f0	3	564.35220
f1	7	573.29890
f2	14	382.03290
f3	18	390.89560

boundary (singular) fit: see? is Singular
[1] 13

	df	AIC
f0	3	594.07660

f1	7	603.87160
f2	15	394.36310
f3	19	404.13700

[1] 14

	df	AIC
f0	3	619.97490
f1	7	630.79520
f2	16	405.88200
f3	20	416.70230

[1] 15

	df	AIC
f0	3	647.16500
f1	7	658.25870
f2	17	423.36470
f3	21	434.45840

> summary(f2)
Linear mixed model fit by REML ['lmerMod']
Formula: temp ~ factor(dpi) + (1 | animal)
Data: body[dpi < i,]
REML criterion at convergence: 389.4
Scaled residuals:

Min	1Q	Median	3Q	Max
-3.6341	-0.4303	0.0747	0.3981	3.8521

Random effects:

Groups	Name	Variance	Std.Dev.
animal	(Intercept)	0.02027	0.1424
Residual		0.15362	0.3919

Number of obs: 345, groups: animal, 23
Fixed effects:

	Estimate	Std. Error	t value
(Intercept)	3.89E+01	8.70E-02	447.334
factor(dpi)1	1.17E+00	1.16E-01	10.119
factor(dpi)2	1.27E+00	1.16E-01	11.022
factor(dpi)3	6.83E-01	1.16E-01	5.906
factor(dpi)4	3.70E-01	1.16E-01	3.198
factor(dpi)5	-7.39E-02	1.16E-01	-0.640
factor(dpi)6	-1.26E-01	1.16E-01	-1.091
factor(dpi)7	-1.00E-01	1.16E-01	-0.865
factor(dpi)8	8.70E-03	1.16E-01	0.075
factor(dpi)9	-5.22E-02	1.16E-01	-0.451
factor(dpi)10	-2.61E-02	1.16E-01	-0.226
factor(dpi)11	-9.57E-02	1.16E-01	-0.828
factor(dpi)12	-7.83E-02	1.16E-01	-0.677
factor(dpi)13	6.05E-13	1.16E-01	0.000
factor(dpi)14	-8.70E-03	1.16E-01	-0.075

Correlation matrix not shown by default, as p = 15 > 12.
Use print(x, correlation=TRUE) or vcov(x) if you need it
> print(f2, correlation=TRUE)
Linear mixed model fit by REML ['lmerMod']
Formula: temp ~ factor(dpi) + (1 | animal)
Data: body[dpi < i,]
REML criterion at convergence: 389.3647
Random effects:

Groups	Name	Std.Dev.
animal	(Intercept)	0.1424
Residual		0.3919

Number of obs: 345, groups: animal, 23

Use print(x, correlation=TRUE) or vcov(x) if you need it

> detach(body)

Supplementary Text ST-4

#############Antibody titres and protective response

```
> data<-read.csv('LVZ194.csv',sep=';')
> data$AbOManisa<-ifelse(data$AbOManisa=='<0.3',0.15,as.numeric(as.character(data$AbOManisa)))
> data$AbO3039<-ifelse(data$AbO3039=='<0.3',0.15,as.numeric(as.character(data$AbO3039)))
> data$AbOAlg<-ifelse(data$AbOAlg=='<0.3',0.15,as.numeric(as.character(data$AbOAlg)))
> data$day<-ifelse(is.na(data$day),'control',data$day)
> data$day<-factor(data$day)
> data$group<-factor(data$group)
> summary(data)
> attach(data)
> table(paste(day,vaccine),group)
```

########## O Manisa serology between groups 21 day and 7 day

```
> t.test(data[group==1|group==3,]$AbOManisa~factor(data[group==1|group==3,]$group),var.equal=T)
Two Sample t-test
data:  data[group == 1 | group == 3, ]$AbOManisa by factor(data[group == 1 | group == 3, ]$group)
t = 3.9598, df = 8, p-value = 0.004178
alternative hypothesis: true difference in means is not equal to 0
95 percent confidence interval:
0.3508227 1.3291773
sample estimates:
mean in group 1  mean in group 3
2.13                1.29
> t.test(data[group==1|group==3,]$AbOAlg~factor(data[group==1|group==3,]$group),var.equal=T)
Two Sample t-test
data:  data[group == 1 | group == 3, ]$AbOAlg by factor(data[group == 1 | group == 3, ]$group)
t = 3.692, df = 8, p-value = 0.006112
alternative hypothesis: true difference in means is not equal to 0
95 percent confidence interval:
0.3491341 1.5108659
sample estimates:
mean in group 1  mean in group 3
2.34                1.41
```

########## O 3039 serology between groups 21 day and 7 day

```
> t.test(data[group==2|group==4,]$AbO3039~factor(data[group==2|group==4,]$group),var.equal=T)
Two Sample t-test
data:  data[group == 2 | group == 4, ]$AbO3039 by factor(data[group == 2 | group == 4, ]$group)
t = 5.8926, df = 8, p-value = 0.0003648
alternative hypothesis: true difference in means is not equal to 0
95 percent confidence interval:
0.4564936 1.0435064
sample estimates:
mean in group 2  mean in group 4
2.76                2.01
> t.test(data[group==2|group==4,]$AbOAlg~factor(data[group==2|group==4,]$group),var.equal=T)
Two Sample t-test
data:  data[group == 2 | group == 4, ]$AbOAlg by factor(data[group == 2 | group == 4, ]$group)
t = 2.245, df = 8, p-value = 0.055
alternative hypothesis: true difference in means is not equal to 0
95 percent confidence interval:
-0.01712081  1.27712081
sample estimates:
mean in group 2  mean in group 4
2.37                1.74
```

########## O Alg serology between groups 21 day and 7 day

```
> t.test(data[group==1|group==2,]$AbOAlg~factor(data[group==1|group==2,]$group),var.equal=T)
Two Sample t-test
```

data: data[group == 1 | group == 2,]$AbOAlg by factor(data[group == 1 | group == 2,]$group)
t = -0.2, df = 8, p-value = 0.8465
alternative hypothesis: true difference in means is not equal to 0
95 percent confidence interval:
-0.3759006 0.3159006
sample estimates:
mean in group 1 mean in group 2
2.34 2.37
> t.test(data[group==3|group==4,]$AbOAlg~factor(data[group==3|group==4,]$group),var.equal=T)
Two Sample t-test
data: data[group == 3 | group == 4,]$AbOAlg by factor(data[group == 3 | group == 4,]$group)
t = -0.95382, df = 8, p-value = 0.3681
alternative hypothesis: true difference in means is not equal to 0
95 percent confidence interval:
-1.1278241 0.4678241
sample estimates:
mean in group 3 mean in group 4
1.41 1.74

### Statistics for Figure 2   #####

########## O Manisa serology between groups 21 day and 7 day (including control)

> summary(aov(data[group!=2&group!=4,]$AbOManisa~data[group!=2&group!=4,]$group))

	Df	Sum Sq	Mean Sq	F value	Pr(>F)	
data[group != 2 & group != 4,]$group	2	6.352	3.176	35.29	2.94E-05	***
Residuals	10	0.9	0.09	---		

Signif. codes: 0 ‘***’ 0.001 ‘**’ 0.01 ‘*’ 0.05 ‘.’ 0.1 ‘ ’ 1
> pairwise.t.test(data[group!=2&group!=4,]$AbOManisa,data[group!=2&group!=4,]$group,pool.sd=F)
Pairwise comparisons using t tests with non-pooled SD
data: data[group != 2 & group != 4,]$AbOManisa and data[group != 2 & group != 4,]$group

	1	3
3	0.015	-
5	4.60E-05	0.015

P value adjustment method: holm
> summary(aov(AbOManisa~group,data[group==1|group==3,]))

	Df	Sum Sq	Mean Sq	F value	Pr(>F)	
group	1	1.764	1.764	15.68	0.00418	**
Residuals	8	0.9	0.1125	---		

Signif. codes: 0 ‘***’ 0.001 ‘**’ 0.01 ‘*’ 0.05 ‘.’ 0.1 ‘ ’ 1
> pairwise.t.test(data[group==1|group==3,]$AbOManisa,data[group==1|group==3,]$group,pool.sd=T)
Pairwise comparisons using t tests with pooled SD
data: data[group == 1 | group == 3,]$AbOManisa and data[group == 1 | group == 3,]$group

	1
3	0.0042

P value adjustment method: holm

########## O 3039 serology between groups 21 day and 7 day (including control)

> summary(aov(data[group!=1&group!=3,]$AbO3039~data[group!=1&group!=3,]$group))

	Df	Sum Sq	Mean Sq	F value	Pr(>F)	
data[group != 1 & group != 3,]$group	2	11.438	5.719	176.5	1.59E-08	***
Residuals	10	0.324	0.032	---		

Signif. codes: 0 ‘***’ 0.001 ‘**’ 0.01 ‘*’ 0.05 ‘.’ 0.1 ‘ ’ 1
> pairwise.t.test(data[group!=1&group!=3,]$AbO3039,data[group!=1&group!=3,]$group,pool.sd=F)
Pairwise comparisons using t tests with non-pooled SD
data: data[group != 1 & group != 3,]$AbO3039 and data[group != 1 & group != 3,]$group

	2	4
4	0.00099	-
5	5.10E-05	2.70E-05

P value adjustment method: holm
> summary(aov(AbO3039~group,data[group==2|group==4,]))

	Df	Sum Sq	Mean Sq	F value	Pr(>F)	
group	1	1.406	1.4062	34.72	0.000365	***
Residuals	8	0.324	0.0405	---		

Signif. codes: 0 '***' 0.001 '**' 0.01 '*' 0.05 '.' 0.1 ' ' 1
> pairwise.t.test(data[group==2|group==4,]$AbO3039,data[group==2|group==4,]$group)
Pairwise comparisons using t tests with pooled SD
data: data[group == 2 | group == 4,]$AbO3039 and data[group == 2 | group == 4,]$group

	2
4	0.00036

P value adjustment method: holm

########## O Alg serology between groups 21 day and 7 day (including control)
> summary(aov(data[group!=2&group!=4,]$AbOAlg~data[group!=2&group!=4,]$group))

	Df	Sum Sq	Mean Sq	F value	Pr(>F)	
data[group != 2 & group != 4,]$group	2	7.529	3.765	29.32	6.56E-05	***
Residuals	10	1.284	0.128	---		

Signif. codes: 0 '***' 0.001 '**' 0.01 '*' 0.05 '.' 0.1 ' ' 1
> pairwise.t.test(data[group!=2&group!=4,]$AbOAlg,data[group!=2&group!=4,]$group,pool.sd=F)
Pairwise comparisons using t tests with non-pooled SD
data: data[group != 2 & group != 4,]$AbOAlg and data[group != 2 & group != 4,]$group

	1	3
3	0.022	-
5	9.70E-07	0.022

P value adjustment method: holm
> summary(aov(AbOAlg~group,data[group==1|group==3,]))

	Df	Sum Sq	Mean Sq	F value	Pr(>F)	
group	1	2.162	2.1622	13.63	0.00611	**
Residuals	8	1.269	0.1586	---		

Signif. codes: 0 '***' 0.001 '**' 0.01 '*' 0.05 '.' 0.1 ' ' 1
> pairwise.t.test(data[group==1|group==3,]$AbOAlg,data[group==1|group==3,]$group,pool.sd=T)
Pairwise comparisons using t tests with pooled SD
data: data[group == 1 | group == 3,]$AbOAlg and data[group == 1 | group == 3,]$group

	1
3	0.0061

P value adjustment method: holm

########## O Algeria serology in O Combo groups
> summary(aov(data[group!=1&group!=3,]$AbOAlg~data[group!=1&group!=3,]$group))

	Df	Sum Sq	Mean Sq	F value	Pr(>F)	
data[group != 1 & group != 3,]$group	2	7.701	3.85	24.22	0.00015	***
Residuals	10	1.59	0.159	---		

Signif. codes: 0 '***' 0.001 '**' 0.01 '*' 0.05 '.' 0.1 ' ' 1
> pairwise.t.test(data[group!=1&group!=3,]$AbOAlg,data[group!=1&group!=3,]$group,pool.sd=F)
Pairwise comparisons using t tests with non-pooled SD
data: data[group != 1 & group != 3,]$AbOAlg and data[group != 1 & group != 3,]$group

	2	4
4	0.06382	-
5	0.00011	0.00807

P value adjustment method: holm

########## O Algeria serology in O3039 groups
> summary(aov(data[group!=2&group!=4,]$AbOAlg~data[group!=2&group!=4,]$group))

	Df	Sum Sq	Mean Sq	F value	Pr(>F)	
data[group != 2 & group != 4,]$group	2	7.529	3.765	29.32	6.56E-05	***

Residuals	10	1.284	0.128	---

Signif. codes: 0 ‘***’ 0.001 ‘**’ 0.01 ‘*’ 0.05 ‘.’ 0.1 ‘ ’ 1

> pairwise.t.test(data[group!=2&group!=4,]$AbOAlg,data[group!=2&group!=4,]$group,pool.sd=F)

Pairwise comparisons using t tests with non-pooled SD

data: data[group != 2 & group != 4,]$AbOAlg and data[group != 2 & group != 4,]$group

	1	3
3	0.022	-
5	9.70E-07	0.022

P value adjustment method: holm

######### O Algeria titers

> tapply(AbOAlg,paste(group,day,vaccine),mean)

1 21 Combo	2 21 O3039	3 7 Combo	4 7 O3039	5 control Control
2.34	2.37	1.41	1.74	0.35

> summary(aov(AbOAlg~group))

	Df	Sum Sq	Mean Sq	F value	Pr(>F)	
group	4	10.121	2.5301	15.93	9.79E-06	***
Residuals	18	2.859	0.1588	---		

Signif. codes: 0 ‘***’ 0.001 ‘**’ 0.01 ‘*’ 0.05 ‘.’ 0.1 ‘ ’ 1

> pairwise.t.test(AbOAlg,group)

Pairwise comparisons using t tests with pooled SD

data: AbOAlg and group

	1	2	3	4
2	0.9066	-	-	-
3	0.0101	0.009	-	-
4	0.0893	0.0893	0.4139	-
5	1.90E-05	1.70E-05	0.0101	0.0012

P value adjustment method: holm

> tapply(AbOAlg,vaccine,mean)

Combo	O3039	Control
1.875	2.055	0.35

> summary(aov(AbOAlg~vaccine))

	Df	Sum Sq	Mean Sq	F value	Pr(>F)	
vaccine	2	6.966	3.483	11.58	0.00046	***
Residuals	20	6.014	0.301	---		

Signif. codes: 0 ‘***’ 0.001 ‘**’ 0.01 ‘*’ 0.05 ‘.’ 0.1 ‘ ’ 1

> pairwise.t.test(AbOAlg,vaccine)

Pairwise comparisons using t tests with pooled SD

data: AbOAlg and vaccine

	Combo	Control
O3039	0.47145	0.00039
Control	0.00083	-

P value adjustment method: holm

> summary(aov(AbOAlg~vaccine+day,data[vaccine!="control",]))

	Df	Sum Sq	Mean Sq	F value	Pr(>F)	
vaccine	2	6.966	3.483	22.27	1.04E-05	***
day	1	3.042	3.042	19.45	0.000301	***
Residuals	19	2.972	0.156	---		

Signif. codes: 0 ‘***’ 0.001 ‘**’ 0.01 ‘*’ 0.05 ‘.’ 0.1 ‘ ’ 1

> summary(aov(AbOAlg~day))

	Df	Sum Sq	Mean Sq	F value	Pr(>F)	
day	2	9.846	4.923	31.42	6.73E-07	***
Residuals	20	3.133	0.157	---		

Signif. codes: 0 ‘***’ 0.001 ‘**’ 0.01 ‘*’ 0.05 ‘.’ 0.1 ‘ ’ 1

> pairwise.t.test(AbOAlg,day)

Pairwise comparisons using t tests with pooled SD

data: AbOAlg and day

	21	7
7	0.00027	-
control	6.30E-07	0.00027

P value adjustment method: holm

######### O3039 titers

> tapply(AbO3039,paste(group,day,vaccine),mean)

1 21 Combo	2 21 O3039	3 7 Combo	4 7 O3039	5 control Control
2.49	2.76	1.95	2.01	0.3

> summary(aov(AbO3039~group))

	Df	Sum Sq	Mean Sq	F value	Pr(>F)	
group	4	12.732	3.183	31.99	5.86E-08	***
Residuals	18	1.791	0.099	---		

Signif. codes: 0 ‘***’ 0.001 ‘**’ 0.01 ‘*’ 0.05 ‘.’ 0.1 ‘ ’ 1

> pairwise.t.test(AbO3039,group)

Pairwise comparisons using t tests with pooled SD

data: AbO3039 and group

	1	2	3	4
2	0.3854	-	-	-
3	0.0578	0.0044	-	-
4	0.0813	0.0072	0.767	-
5	1.70E-07	3.20E-08	8.00E-06	5.60E-06

P value adjustment method: holm

> tapply(AbO3039,vaccine,mean)

Combo	O3039	Control
2.22	2.39	0.30

> summary(aov(AbO3039~vaccine))

	Df	Sum Sq	Mean Sq	F value	Pr(>F)	
vaccine	2	10.597	5.299	26.99	2.09E-06	***
Residuals	20	3.926	0.196	---		

Signif. codes: 0 ‘***’ 0.001 ‘**’ 0.01 ‘*’ 0.05 ‘.’ 0.1 ‘ ’ 1

> pairwise.t.test(AbO3039,vaccine)

Pairwise comparisons using t tests with pooled SD

data: AbO3039 and vaccine

	Combo	Control
O3039	0.41	1.90E-06
Control	4.10E-06	-

P value adjustment method: holm

> summary(aov(AbO3039~vaccine+day,data[vaccine!="control",]))

	Df	Sum Sq	Mean Sq	F value	Pr(>F)	
vaccine	2	10.597	5.299	54.53	1.34E-08	***
day	1	2.08	2.08	21.41	0.00018	***
Residuals	19	1.846	0.097	---		

Signif. codes: 0 ‘***’ 0.001 ‘**’ 0.01 ‘*’ 0.05 ‘.’ 0.1 ‘ ’ 1

> summary(aov(AbO3039~day))

	Df	Sum Sq	Mean Sq	F value	Pr(>F)	
day	2	12.541	6.271	63.27	2.24E-09	***
Residuals	20	1.982	0.099	---		

Signif. codes: 0 ‘***’ 0.001 ‘**’ 0.01 ‘*’ 0.05 ‘.’ 0.1 ‘ ’ 1

> pairwise.t.test(AbO3039,day)

Pairwise comparisons using t tests with pooled SD

data: AbO3039 and day

	21	7
7	0.00018	-
control	1.30E-09	1.90E-07

P value adjustment method: holm

########### O Manisa titers

> tapply(AbOManisa,paste(group,day,vaccine),mean)

1 21 Combo	2 21 O3039	3 7 Combo	4 7 O3039	5 control Control
2.13	2.01	1.29	1.29	0.30

> summary(aov(AbOManisa~group))

	Df	Sum Sq	Mean Sq	F value	Pr(>F)	
group	4	8.046	2.011	21.63	1.13E-06	***
Residuals	18	1.674	0.093	---		

Signif. codes: 0 ‘***’ 0.001 ‘**’ 0.01 ‘*’ 0.05 ‘.’ 0.1 ‘ ’ 1

> pairwise.t.test(AbOManisa,group)

Pairwise comparisons using t tests with pooled SD

data: AbOManisa and group

	1	2	3	4
2	1	-	-	-
3	0.0025	0.0061	-	-
4	0.0025	0.0061	1	-
5	1.70E-06	3.90E-06	0.0025	0.0025

P value adjustment method: holm

> tapply(AbOManisa,vaccine,mean)

Combo	O3039	Control
1.71	1.65	0.30

> summary(aov(AbOManisa~vaccine))

	Df	Sum Sq	Mean Sq	F value	Pr(>F)	
vaccine	2	4.986	2.493	10.53	0.00075	***
Residuals	20	4.734	0.2367	---		

Signif. codes: 0 ‘***’ 0.001 ‘**’ 0.01 ‘*’ 0.05 ‘.’ 0.1 ‘ ’ 1

> pairwise.t.test(AbOManisa,vaccine)

Pairwise comparisons using t tests with pooled SD

data: AbOManisa and vaccine

	Combo	Control
O3039	0.78556	0.00085
Control	0.00082	-

P value adjustment method: holm

> summary(aov(AbOManisa~vaccine+day,data[vaccine!="control",]))

	Df	Sum Sq	Mean Sq	F value	Pr(>F)	
vaccine	2	4.986	2.493	28	2.17E-06	***
day	1	3.042	3.042	34.16	1.25E-05	***
Residuals	19	1.692	0.0891	---		

Signif. codes: 0 ‘***’ 0.001 ‘**’ 0.01 ‘*’ 0.05 ‘.’ 0.1 ‘ ’ 1

> summary(aov(AbOManisa~day))

	Df	Sum Sq	Mean Sq	F value	Pr(>F)	
day	2	8.01	4.005	46.84	2.84E-08	***
Residuals	20	1.71	0.086	---		

Signif. codes: 0 ‘***’ 0.001 ‘**’ 0.01 ‘*’ 0.05 ‘.’ 0.1 ‘ ’ 1

> pairwise.t.test(AbOManisa,day)

Pairwise comparisons using t tests with pooled SD

data: AbOManisa and day

	21	7
7	1.60E-05	-
control	3.80E-08	4.90E-05

P value adjustment method: holm

###############Linear Models Ab O3039 #############

> q0<-lm(AbO3039~1,data[vaccine!="control",])

> q1<-lm(AbO3039~day,data[vaccine!="control",])

> q2<-lm(AbO3039~vaccine,data[vaccine!="control",])

> AIC(q0,q1,q2)

	df	AIC

q0	2	58.69709
q1	4	16.89215
q2	4	32.61156

> q3<-lm(AbO3039~day+vaccine,data[vaccine!="control",])
> AIC(q1,q3)

	df	AIC
q1	4	16.89215
q3	5	17.25585

> q4<-lm(AbO3039~day+vaccine+day:vaccine,data[vaccine!="control",])
> AIC(q1,q4)

	df	AIC
q1	4	16.89215
q4	6	18.55861

> summary(q1)
Call:
lm(formula = AbO3039 ~ day, data = data[vaccine != "control",])
Residuals:

Min	1Q	Median	3Q	Max
-0.930	-0.203	0.000	0.120	0.420

Coefficients:

	Estimate	Std. Error	t value	Pr(>|t|)	
(Intercept)	2.625	0.09956	26.367	< 2e-16	***
day7	-0.645	0.14079	-4.581	0.000181	***
daycontrol	-2.325	0.20724	-11.219	4.42E-10	***

Signif. codes: 0 ‘***’ 0.001 ‘**’ 0.01 ‘*’ 0.05 ‘.’ 0.1 ‘ ’ 1
Residual standard error: 0.3148 on 20 degrees of freedom
Multiple R-squared: 0.8635,	Adjusted R-squared: 0.8499
F-statistic: 63.27 on 2 and 20 DF, p-value: 2.244e-09

###############Linear Models Ab OManisa
> q0<-lm(AbOManisa~1,data[vaccine!="control",])
> q1<-lm(AbOManisa~day,data[vaccine!="control",])
> q2<-lm(AbOManisa~vaccine,data[vaccine!="control",])
> AIC(q0,q1,q2)

	df	AIC
q0	2	49.46107
q1	4	13.49415
q2	4	36.91453

> q3<-lm(AbOManisa~day+vaccine,data[vaccine!="control",])
> AIC(q1,q3)

	df	AIC
q1	4	13.49415
q3	5	15.25076

> q4<-lm(AbOManisa~day+vaccine+day:vaccine,data[vaccine!="control",])
> AIC(q1,q4)

	df	AIC
q1	4	13.49415
q4	6	17.00477

> summary(q1)
Call:
lm(formula = AbOManisa ~ day, data = data[vaccine != "control",])
Residuals:

Min	1Q	Median	3Q	Max
-0.54	-0.12	0.03	0.20	0.51

Coefficients:

	Estimate	Std. Error	t value	Pr(>|t|)	
(Intercept)	2.07	0.09247	22.387	1.24E-15	***
day7	-0.78	0.13077	-5.965	7.83E-06	***
daycontrol	-1.77	0.19248	-9.196	1.27E-08	***

Signif. codes: 0 '***' 0.001 '**' 0.01 '*' 0.05 '.' 0.1 ' ' 1
Residual standard error: 0.2924 on 20 degrees of freedom
Multiple R-squared: 0.8241, Adjusted R-squared: 0.8065
F-statistic: 46.84 on 2 and 20 DF, p-value: 2.84e-08

###############Linear Models Ab O Algeria

```
> > q0<-lm(AbOAlg~1,data[vaccine!="control",])
> q1<-lm(AbOAlg~day,data[vaccine!="control",])
> q2<-lm(AbOAlg~vaccine,data[vaccine!="control",])
> AIC(q0,q1,q2)
```

	df	AIC
q0	2	56.11246
q1	4	27.42427
q2	4	42.41697

```
> q3<-lm(AbOAlg~day+vaccine,data[vaccine!="control",])
> AIC(q1,q3)
```

	df	AIC
q1	4	27.42427
q3	5	28.20334

```
> q4<-lm(AbOAlg~day+vaccine+day:vaccine,data[vaccine!="control",])
> AIC(q1,q4)
```

	df	AIC
q1	4	27.42427
q4	6	29.31566

```
> summary(q1)
```

Call:
lm(formula = AbOAlg ~ day, data = data[vaccine != "control",])
Residuals:

Min	1Q	Median	3Q	Max
-0.975	-0.225	0.045	0.0875	0.825

Coefficients:

	Estimate	Std. Error	t value	Pr(>|t|)	
(Intercept)	2.355	0.1252	18.814	3.46E-14	***
day7	-0.78	0.177	-4.406	0.000272	***
daycontrol	-2.005	0.2606	-7.695	2.11E-07	***

Signif. codes: 0 '***' 0.001 '**' 0.01 '*' 0.05 '.' 0.1 ' ' 1

Residual standard error: 0.3958 on 20 degrees of freedom
Multiple R-squared: 0.7586, Adjusted R-squared: 0.7344
F-statistic: 31.42 on 2 and 20 DF, p-value: 6.725e-07

Supplementary Text ST-5
######### NSP antibodies ###########
############ NSP response between groups ##########

> data<-read.csv('LVZ194.csv',sep=';')
> table(paste(vaccine,day),group)
> tapply(nsp0,group,summary)
> summary(aov(nsp0~group))

	Df	Sum Sq	Mean Sq	F value	Pr(>F)	
group	4	408.3	102.08	1.353	0.289	
Residuals	18	1358.4	75.47	----		

> tapply(nsp5,group,summary)
> summary(aov(nsp5~group))

	Df	Sum Sq	Mean Sq	F value	Pr(>F)	
group	4	6.41E+02	160.34	2.138	0.118	
Residuals	18	1349.7	74.98			

> tapply(nsp11,group,summary)
> summary(aov(nsp11~group))

	Df	Sum Sq	Mean Sq	F value	Pr(>F)	
group	4	4.12E+02	102.9	3.397	0.0309	*
Residuals	18	545.4	30.3	----		

Signif. codes: 0 ‘***’ 0.001 ‘**’ 0.01 ‘*’ 0.05 ‘.’ 0.1 ‘ ’ 1
> pairwise.t.test(nsp11,group) # No pairwise difference
Pairwise comparisons using t tests with pooled SD
data: nsp11 and group

	1	2	3	4
2	1.000	-	-	-
3	0.578	0.998	-	-
4	0.998	0.494	0.053	-
5	0.998	0.578	0.100	1.000

P value adjustment method: holm
> tapply(nsp14,group,summary)
> summary(aov(nsp14~group))

	Df	Sum Sq	Mean Sq	F value	Pr(>F)	
group	4	544	136	4.583	0.00997	**
Residuals	18	534.2	29.68	---		

Signif. codes: 0 ‘***’ 0.001 ‘**’ 0.01 ‘*’ 0.05 ‘.’ 0.1 ‘ ’ 1
> pairwise.t.test(nsp14,group)
Pairwise comparisons using t tests with pooled SD
data: nsp14 and group

	1	2	3	4
2	1.000	-	-	-
3	0.109	0.527	-	-
4	1.000	0.482	0.016	-
5	1.000	0.527	0.033	1.000

P value adjustment method: holm
> tapply(nsp21,group,summary)
> summary(aov(nsp21~group))

	Df	Sum Sq	Mean Sq	F value	Pr(>F)	
group	4	343.7	85.94	4.597	0.00984	**
Residuals	18	336.5	18.7	---		

Signif. codes: 0 ‘***’ 0.001 ‘**’ 0.01 ‘*’ 0.05 ‘.’ 0.1 ‘ ’ 1
> pairwise.t.test(nsp21,group) # No pairwise difference
Pairwise comparisons using t tests with pooled SD
data: nsp21 and group

	1	2	3	4
2	0.673	-	-	-
3	0.602	1.000	-	-

4	0.673	0.066	0.051	-
5	0.673	0.076	0.066	1.000

P value adjustment method: holm
> tapply(nsp28,group,summary)
> summary(aov(nsp28~group))

	Df	Sum Sq	Mean Sq	F value	Pr(>F)	
group	4	200.6	50.14	6.128	0.00271	**
Residuals	18	147.3	8.18	---		

Signif. codes: 0 ‘***’ 0.001 ‘**’ 0.01 ‘*’ 0.05 ‘.’ 0.1 ‘ ’ 1
> pairwise.t.test(nsp28,group)
Pairwise comparisons using t tests with pooled SD
data: nsp28 and group

	1	2	3	4
2	0.213	-	-	-
3	0.688	0.628	-	-
4	0.576	0.012	0.156	-
5	0.213	0.006	0.058	0.688

P value adjustment method: holm
> tapply(nsp33,group,summary)
> summary(aov(nsp33~group))

	Df	Sum Sq	Mean Sq	F value	Pr(>F)	
group	4	112.17	28.041	6.474	0.00207	**
Residuals	18	77.96	4.331			

Signif. codes: 0 ‘***’ 0.001 ‘**’ 0.01 ‘*’ 0.05 ‘.’ 0.1 ‘ ’ 1
> pairwise.t.test(nsp33,group)
Pairwise comparisons using t tests with pooled SD
data: nsp33 and group

	1	2	3	4
2	0.0938	-	-	-
3	0.8397	0.0689	-	-
4	0.7592	0.0060	0.7867	-
5	0.2972	0.0029	0.3489	0.7867

P value adjustment method: holm

> ########### NSP response between vaccines ##########
> tapply(nsp0,vaccine,summary)
> summary(aov(nsp0~vaccine))

	Df	Sum Sq	Mean Sq	F value	Pr(>F)
vaccine	2	55.700	27.84	0.325	0.726
Residuals	20	1711.000	85.55		

> tapply(nsp5,vaccine,summary)
> summary(aov(nsp5~vaccine))

	Df	Sum Sq	Mean Sq	F value	Pr(>F)
vaccine	2	121.7	60.87	0.651	0.532
Residuals	20	1869.3	93.47		

> tapply(nsp11,vaccine,summary)
> summary(aov(nsp11~vaccine))

	Df	Sum Sq	Mean Sq	F value	Pr(>F)
vaccine	2	189	94.5	2.46	0.111
Residuals	20	768.1	38.41		

> tapply(nsp14,vaccine,summary)
> summary(aov(nsp14~vaccine))

	Df	Sum Sq	Mean Sq	F value	Pr(>F)
vaccine	2	210.7	105.36	2.429	0.114
Residuals	20	867.5	43.37		

> tapply(nsp21,vaccine,summary)

> summary(aov(nsp21~vaccine))

	Df	Sum Sq	Mean Sq	F value	Pr(>F)
vaccine	2	116.6	58.28	2.068	0.153
Residuals	20	563.7	28.18		

> tapply(nsp28,vaccine,summary)
> summary(aov(nsp28~vaccine))

	Df	Sum Sq	Mean Sq	F value	Pr(>F)
vaccine	2	74.63	37.31	2.731	0.894
Residuals	20	273.23	13.66	---	

Signif. codes: 0 ‘***’ 0.001 ‘**’ 0.01 ‘*’ 0.05 ‘.’ 0.1 ‘ ’ 1
> tapply(nsp33,vaccine,summary)
> summary(aov(nsp33~vaccine))

	Df	Sum Sq	Mean Sq	F value	Pr(>F)
vaccine	2	39	19.501	2.581	0.101
Residuals	20	151.1	7.556		

> ########### NSP response between day of challenge ##########
> tapply(nsp0,day,summary)
> summary(aov(nsp0~day))

	Df	Sum Sq	Mean Sq	F value	Pr(>F)
day	2	362.1	181.06	2.578	0.101
Residuals	20	1404.6	70.23		

> tapply(nsp5,day,summary)
> summary(aov(nsp5~day))

	Df	Sum Sq	Mean Sq	F value	Pr(>F)	
day	2	526.6	263.31	3.596	0.0463	*
Residuals	20	1464.5	73.22	----		

Signif. codes: 0 ‘***’ 0.001 ‘**’ 0.01 ‘*’ 0.05 ‘.’ 0.1 ‘ ’ 1
> pairwise.t.test(nsp5,day) # No pairwise difference
Pairwise comparisons using t tests with pooled SD
data: nsp5 and day

	21	7
7	0.07	-
Control	0.87	0.16

P value adjustment method: holm
> tapply(nsp11,day,summary)
> summary(aov(nsp11~day))

	Df	Sum Sq	Mean Sq	F value	Pr(>F)
day	2	94	46.99	1.089	0.356
Residuals	20	863.1	43.16		

> tapply(nsp14,day,summary)
> summary(aov(nsp14~day))

	Df	Sum Sq	Mean Sq	F value	Pr(>F)
day	2.00	109.70	54.83	1.13	0.342
Residuals	20.00	968.50	48.43		

> pairwise.t.test(nsp14,day)
Pairwise comparisons using t tests with pooled SD
data: nsp14 and day

	21	7
7	0.66	-
control	0.49	0.45

P value adjustment method: holm
> tapply(nsp21,day,summary)
> summary(aov(nsp21~day))

	Df	Sum Sq	Mean Sq	F value	Pr(>F)
day	2.00	107.50	53.76	1.88	0.179
Residuals	20.00	572.70	28.64		

> pairwise.t.test(nsp21,day)
Pairwise comparisons using t tests with pooled SD
data: nsp21 and day

	21	7
7	0.47	-
control	0.20	0.33

P value adjustment method: holm
> tapply(nsp28,day,summary)
> summary(aov(nsp28~day))

	Df	Sum Sq	Mean Sq	F value	Pr(>F)	
day	2	107.2	53.59	4.453	0.0252	*
Residuals	20	240.7	12.03	----		

Signif. codes: 0 ‘***’ 0.001 ‘**’ 0.01 ‘*’ 0.05 ‘.’ 0.1 ‘ ’ 1
> pairwise.t.test(nsp28,day)
Pairwise comparisons using t tests with pooled SD
data: nsp28 and day

	21	7
7	0.181	-
control	0.027	0.181

P value adjustment method: holm
> tapply(nsp33,day,summary)
> summary(aov(nsp33~day))

	Df	Sum Sq	Mean Sq	F value	Pr(>F)	
day	2	73.79	36.9	6.343	0.00736	**
Residuals	20	116.33	5.82	----		

Signif. codes: 0 ‘***’ 0.001 ‘**’ 0.01 ‘*’ 0.05 ‘.’ 0.1 ‘ ’ 1
> pairwise.t.test(nsp33,day)
Pairwise comparisons using t tests with pooled SD
data: nsp33 and day

	21	7
7	0.032	-
control	0.015	0.187

P value adjustment method: holm

########## linear and linear mixed models for NSP antibodies ##############
> ns<-read.csv("nsp.csv",sep=';')
> ns$vac<-factor(substr(ns$vactype,1,4))
> attach(ns)
> summary(ns)
> tapply(percinh,animal,length)
> h0<-lmer(percinh~(1|animal))
boundary (singular) fit: see ?isSingular
> h1<-lmer(percinh~factor(dpv)+(1|animal))
boundary (singular) fit: see ?isSingular
> h2<-lmer(percinh~vactype+(1|animal))
boundary (singular) fit: see ?isSingular
> h2a<-lmer(percinh~vac+(1|animal))
boundary (singular) fit: see ?isSingular
> h3<-lmer(percinh~prot+(1|animal))
boundary (singular) fit: see ?isSingular
> h4<-lmer(percinh~factor(dpc)+(1|animal))
> AIC(h0,h1,h2,h2a,h3,h4)

	df	AIC
h0	3	1491.296
h1	5	1484.43
h2	7	1475.986
h2a	5	1484.822

h3	4	1487.966
h4	9	1025.289

> summary(h4)   #I do not understand the singularity we have 7 observations per animal. but we ignore it as model h4 does not give this warning
Linear mixed model fit by REML ['lmerMod']
Formula: percinh ~ factor(dpc) + (1 | animal)
REML criterion at convergence: 1007.3
Scaled residuals:

Min	1Q	Median	3Q	Max
-3.00231	-0.54037	0.04811	0.53723	2.8935

Random effects:

Groups	Name	Variance	Std.Dev.
animal	(Intercept)	18.01	4.244
Residual		27.52	5.246

Number of obs: 161, groups: animal, 23
Fixed effects:

	Estimate	Std. Error	t value
(Intercept)	33.364	1.407	23.714
factor(dpc)5	7.407	1.547	4.789
factor(dpc)11	54.06	1.547	34.949
factor(dpc)14	55.002	1.547	35.558
factor(dpc)21	54.674	1.547	35.345
factor(dpc)28	56.854	1.547	36.755
factor(dpc)33	59.007	1.547	38.147

Correlation of Fixed Effects:

	(Intr)	fct()5	fc()11	fc()14	fc()21	fc()28
factr(dpc)5	-0.55					
fctr(dpc)11	-0.55	0.50				
fctr(dpc)14	-0.55	0.50	0.50			
fctr(dpc)21	-0.55	0.50	0.50	0.50		
fctr(dpc)28	-0.55	0.50	0.50	0.50	0.50	
fctr(dpc)33	-0.55	0.50	0.50	0.50	0.50	0.50

> h5<-lmer(percinh~factor(dpc)+factor(dpv)+(1|animal))
> h6<-lmer(percinh~factor(dpc)+vactype+(1|animal))
> h6a<-lmer(percinh~factor(dpc)+vac+(1|animal))
> h7<-lmer(percinh~factor(dpc)+prot+(1|animal))
> AIC(h4,h5,h6,h6a,h7)

	df	AIC
h4	9	1025.289
h5	11	1018.709
h6	13	1006.9
h6a	11	1020.319
h7	10	1021.786

> summary(h6)
Linear mixed model fit by REML ['lmerMod']
Formula: percinh ~ factor(dpc) + vactype + (1 | animal)
REML criterion at convergence: 980.9
Scaled residuals:

Min	1Q	Median	3Q	Max
-3.01572	-0.52373	0.04974	0.50303	2.88008

Random effects:

Groups	Name	Variance	Std.Dev.
animal	(Intercept)	10.87	3.297
Residual		27.52	5.246

Number of obs: 161, groups: animal, 23
Fixed effects:

	Estimate	Std. Error	t value
(Intercept)	32.826	1.997	16.442
factor(dpc)5	7.407	1.547	4.789
factor(dpc)11	54.06	1.547	34.949
factor(dpc)14	55.002	1.547	35.558
factor(dpc)21	54.674	1.547	35.345
factor(dpc)28	56.854	1.547	36.755
factor(dpc)33	59.007	1.547	38.147
vactypecomb7	-1.629	2.433	-0.669
vactypecontr	2.662	2.81	0.947
vactypeO303921	-2.904	2.433	-1.193
vactypeO30397	5.408	2.433	2.222

Correlation of Fixed Effects:

	(Intr)	fct()5	fc()11	fc()14	fc()21	fc()28	fc()33	vctyp7	vctypc	vO303921
factr(dpc)5	-0.387									
fctr(dpc)11	-0.387	0.500								
fctr(dpc)14	-0.387	0.500	0.500							
fctr(dpc)21	-0.387	0.500	0.500	0.500						
fctr(dpc)28	-0.387	0.500	0.500	0.500	0.500					
fctr(dpc)33	-0.387	0.500	0.500	0.500	0.500	0.500				
vactypecmb7	-0.609	0.000	0.000	0.000	0.000	0.000	0.000			
vactypecntr	-0.528	0.000	0.000	0.000	0.000	0.000	0.000	0.433		
vctyO303921	-0.609	0.000	0.000	0.000	0.000	0.000	0.000	0.500	0.433	
vctypO30397	-0.609	0.000	0.000	0.000	0.000	0.000	0.000	0.500	0.433	0.500

```
> h8<-lmer(percinh~factor(dpc)+vactype+factor(dpv)+(1|animal))  #nonsense I know dpv is part of vactype
fixed-effect model matrix is rank deficient so dropping 2 columns / coefficients
> h9<-lmer(percinh~factor(dpc)+vactype+vactype:factor(dpc)+(1|animal))
> h10<-lmer(percinh~factor(dpc)+vactype+prot+(1|animal))
> h11<-lmer(percinh~factor(dpc)+vactype+vac+(1|animal)) #nonsense I know vac is part of vactype
fixed-effect model matrix is rank deficient so dropping 2 columns / coefficients
> AIC(h6,h8,h9,h10,h11)
```

	df	AIC
h6	13	1006.9004
h8	13	1006.9004
h9	37	906.5031
h10	14	1005.0319
h11	13	1006.9004

```
> h11<-lmer(percinh~factor(dpc)+vactype+vactype:factor(dpc)+factor(dpv)+(1|animal))
fixed-effect model matrix is rank deficient so dropping 2 columns / coefficients
> h12<-lmer(percinh~factor(dpc)+vactype+vactype:factor(dpc)+prot+(1|animal))
> h13<-lmer(percinh~factor(dpc)+vactype+vactype:factor(dpc)+vac+(1|animal))
fixed-effect model matrix is rank deficient so dropping 2 columns / coefficients
> AIC(h9,h11,h12,h13)
```

	df	AIC
h9	37	906.5031
h11	37	906.5031
h12	38	904.6345
h13	37	906.5031

```
> summary(h12)  #difference in AIC less than 2 so not truly significant  t-value also low.
Linear mixed model fit by REML ['lmerMod']
Formula: percinh ~ factor(dpc) + vactype + vactype:factor(dpc) + prot +      (1 | animal)
REML criterion at convergence: 828.6
Scaled residuals:
```

Min	1Q	Median	3Q	Max
-2.42664	-0.43492	0.02643	0.39563	2.32534

Random effects:

Groups	Name	Variance	Std.Dev.
animal	(Intercept)	12.39	3.52
Residual		23	4.796

Number of obs: 161, groups: animal, 23

Fixed effects:

	Estimate	Std. Error	t value
(Intercept)	30.77792	3.86199	7.969
factor(dpc)5	6.68253	3.03327	2.203
factor(dpc)11	56.95039	3.03327	18.775
factor(dpc)14	59.21074	3.03327	19.520
factor(dpc)21	57.81267	3.03327	19.060
factor(dpc)28	59.58922	3.03327	19.645
factor(dpc)33	61.58045	3.03327	20.302
vactypecomb7	5.15046	3.80387	1.354
vactypecontr	-1.13330	5.16832	-0.219
vactypeO303921	-1.50405	3.76244	-0.400
vactypeO30397	9.16337	3.92554	2.334
prot	-0.06873	2.79947	-0.025
factor(dpc)5:vactypecomb7	0.32366	4.28969	0.075
factor(dpc)11:vactypecomb7	-11.56549	4.28969	-2.696
factor(dpc)14:vactypecomb7	-14.58997	4.28969	-3.401
factor(dpc)21:vactypecomb7	-9.90166	4.28969	-2.308
factor(dpc)28:vactypecomb7	-6.92225	4.28969	-1.614
factor(dpc)33:vactypecomb7	-4.89406	4.28969	-1.141
factor(dpc)5:vactypecontr	-0.48700	4.95331	-0.098
factor(dpc)11:vactypecontr	6.03826	4.95331	1.219
factor(dpc)14:vactypecontr	4.89898	4.95331	0.989
factor(dpc)21:vactypecontr	5.74188	4.95331	1.159
factor(dpc)28:vactypecontr	5.62888	4.95331	1.136
factor(dpc)33:vactypecontr	4.26504	4.95331	0.861
factor(dpc)5:vactypeO303921	0.33317	4.28969	0.078
factor(dpc)11:vactypeO303921	-0.79749	4.28969	-0.186
factor(dpc)14:vactypeO303921	-1.84073	4.28969	-0.429
factor(dpc)21:vactypeO303921	-2.78002	4.28969	-0.648
factor(dpc)28:vactypeO303921	-2.60884	4.28969	-0.608
factor(dpc)33:vactypeO303921	-2.10534	4.28969	-0.491
factor(dpc)5:vactypeO30397	2.97001	4.28969	0.692
factor(dpc)11:vactypeO30397	-4.55577	4.28969	-1.062
factor(dpc)14:vactypeO30397	-5.86707	4.28969	-1.368
factor(dpc)21:vactypeO30397	-5.20289	4.28969	-1.213
factor(dpc)28:vactypeO30397	-6.42695	4.28969	-1.498
factor(dpc)33:vactypeO30397	-7.39724	4.28969	-1.724

Correlation matrix not shown by default, as p = 36 > 12.
Use print(x, correlation=TRUE) or vcov(x) if you need it
> summary(h9) #The model indicates a lower percinh in comb7 than in comb21 especially on day 11, 14 and 21
Linear mixed model fit by REML ['lmerMod']
Formula: percinh ~ factor(dpc) + vactype + vactype:factor(dpc) + (1 | animal)
REML criterion at convergence: 832.5
Scaled residuals:

Min	1Q	Median	3Q	Max
-2.43159	-0.44916	0.02033	0.39049	2.33132

Random effects:

Groups	Name	Variance	Std.Dev.
animal	(Intercept)	11.52	3.394
Residual		23.00	4.796

Number of obs: 161, groups: animal, 23
Fixed effects:

	Estimate	Std. Error	t value
(Intercept)	30.7092	2.6275	11.687
factor(dpc)5	6.6825	3.0333	2.203
factor(dpc)11	56.9504	3.0333	18.775
factor(dpc)14	59.2107	3.0333	19.52
factor(dpc)21	57.8127	3.0333	19.06
factor(dpc)28	59.5892	3.0333	19.645
factor(dpc)33	61.5805	3.0333	20.302
vactypecomb7	5.1642	3.7159	1.39
vactypecontr	-1.0646	4.2907	-0.248
vactypeO303921	-1.5040	3.7159	-0.405
vactypeO30397	9.1909	3.7159	2.473
factor(dpc)5:vactypecomb7	0.3237	4.2897	0.075
factor(dpc)11:vactypecomb7	-11.5655	4.2897	-2.696
factor(dpc)14:vactypecomb7	-14.5900	4.2897	-3.401
factor(dpc)21:vactypecomb7	-9.9017	4.2897	-2.308
factor(dpc)28:vactypecomb7	-6.9222	4.2897	-1.614
factor(dpc)33:vactypecomb7	-4.8941	4.2897	-1.141
factor(dpc)5:vactypecontr	-0.4870	4.9533	-0.098
factor(dpc)11:vactypecontr	6.0383	4.9533	1.219
factor(dpc)14:vactypecontr	4.8990	4.9533	0.989
factor(dpc)21:vactypecontr	5.7419	4.9533	1.159
factor(dpc)28:vactypecontr	5.6289	4.9533	1.136
factor(dpc)33:vactypecontr	4.2650	4.9533	0.861
factor(dpc)5:vactypeO303921	0.3332	4.2897	0.078
factor(dpc)11:vactypeO303921	-0.7975	4.2897	-0.186
factor(dpc)14:vactypeO303921	-1.8407	4.2897	-0.429
factor(dpc)21:vactypeO303921	-2.7800	4.2897	-0.648
factor(dpc)28:vactypeO303921	-2.6088	4.2897	-0.608
factor(dpc)33:vactypeO303921	-2.1053	4.2897	-0.491
factor(dpc)5:vactypeO30397	2.9700	4.2897	0.692
factor(dpc)11:vactypeO30397	-4.5558	4.2897	-1.062
factor(dpc)14:vactypeO30397	-5.8671	4.2897	-1.368
factor(dpc)21:vactypeO30397	-5.2029	4.2897	-1.213
factor(dpc)28:vactypeO30397	-6.4269	4.2897	-1.498
factor(dpc)33:vactypeO30397	-7.3972	4.2897	-1.724

Correlation matrix not shown by default, as p = 35 > 12.
Use print(x, correlation=TRUE) or vcov(x) if you need it
> detach(ns)
